# Bilateral collagenous fibroma of the hard palate: a case report and review of the literature

**DOI:** 10.1186/s13256-022-03691-2

**Published:** 2023-01-07

**Authors:** Hagar A. El-naggar, Yehia A. El-Mahallawy, Mohamed I. Harby, Nourhan A. Abou Madawi

**Affiliations:** 1 Oral Pathology Department, Faculty of Dentistry, Alexandria University, Champollion Street, Azarita, 21521 Alexandria Egypt; 2 Oral and Maxillofacial Surgery Department, Faculty of Dentistry, Alexandria University, Alexandria, Egypt

**Keywords:** Collagenous fibroma, Desmoplastic fibroblastoma, Oral cavity, Fibrous tumor, Bilateral

## Abstract

**Background:**

Collagenous fibroma or desmoplastic fibroblastoma is a rare benign fibrous tissue tumor. It usually presents as a painless, slowly growing mass. Collagenous fibroma arises ordinarily inside the subcutaneous tissues or skeletal muscles. Histopathologically, the tumor consists of scattered stellate and spindle cells in a hypovascular collagenous stroma without atypia or infiltration. The oral cavity is a very uncommon site for desmoplastic fibroblastoma. Only 15 published articles in the literature reported the intraoral location. We present a case of collagenous fibroma with a bilateral distribution on the hard palate. This is the second case of bilateral collagenous fibroma after a previously reported one in literature; however, our case was larger, occupying almost the whole palate. We discuss the management of this rare tumor and how we can reach definite diagnosis.

**Case presentation:**

A 37-year-old Caucasian female patient had a huge bilateral firm palatal mass that caused breathing problems. There was no history of trauma and the patient had no relevant medical history Total surgical excision under general anesthesia was carried out and histopathological examination suggested a benign mesenchymal tumor. Immunohistochemistry was necessary to confirm the tumor origin and to exclude aggressive fibromatosis. A diagnosis of bilateral collagenous fibroma was reached. Six months after surgery, there was no recurring lesion and the patient’s health was good.

**Conclusions:**

Collagenous fibroma is a benign fibrous tissue tumor of unknown cause that is treated with simple excision. The prognosis is good with no recurrence. Reaching an accurate diagnosis is mandatory to avoid aggressive treatment since collagenous fibroma may be misdiagnosed as aggressive fibromatosis in case of massive size. Clinicians and pathologists should be aware of this unusual tumor for conservative management without side effects.

## Introduction

Collagenous fibroma or desmoplastic fibroblastoma is a rare benign soft tissue tumor that can occur in different body sites including arms, shoulders, legs, hands, and feet [1]. It is a hypocellular tumor of fibroblastic/myofibroblastic origin [2]. Males are more affected with peak incidence in the sixth decade [1]. The tumor mostly arises from the subcutaneous tissue, which explains its rare occurrence in the oral cavity [3]. It usually presents as a painless, firm, slowly growing mass [4]. Treatment is by surgical excision with no evidence of recurrence [2].

Collagenous fibroma was presented bilaterally for the first time as reported by Vasconcelos *et al*. [5]. We present a case of bilateral collagenous fibroma of the hard palate. This is the first time we encounter collagenous fibroma in our department.

## Case presentation

### Chief complaint

A 37-year-old Caucasian female patient sought treatment at the Faculty of Dentistry, Alexandria University because of a large palatal lesion causing difficulty in breathing. She was referred to the Oral and Maxillofacial Surgery Department of our faculty.

### Medical history

No relevant medical history.

#### Dental history

The patient had extracted upper right first and second premolars.

#### Clinical examination

**Fig. 1:**
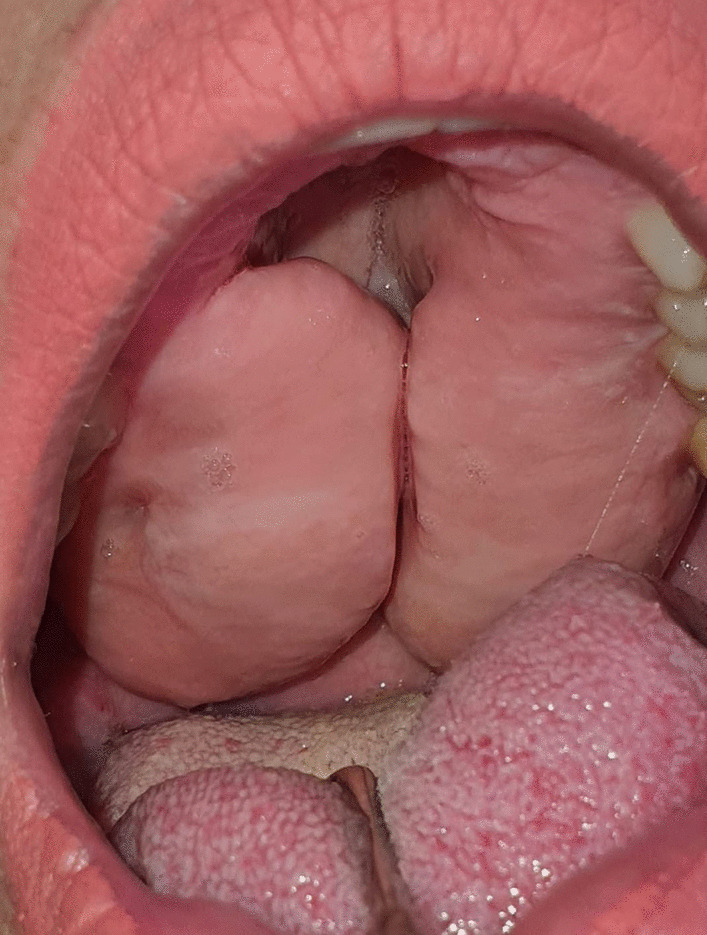
Intraoral view showing a bilateral lesion in the hard palate covered by normal mucosa

The lesion was bilateral, presented on the hard palate, about 5 × 2 cm each and started to appear 10 years ago. It was pedunculated, firm in consistency with normal overlying mucosa (Fig. 1). The patient did not mention any history of trauma and there was no pain. Differential diagnosis included peripheral giant cell granuloma, odontogenic myxoma, neurofibroma, minor salivary gland tumor, and desmoid tumor.

#### Radiological examination

There was no bone invasion.

#### Provisional diagnosis management

**Fig. 2:**
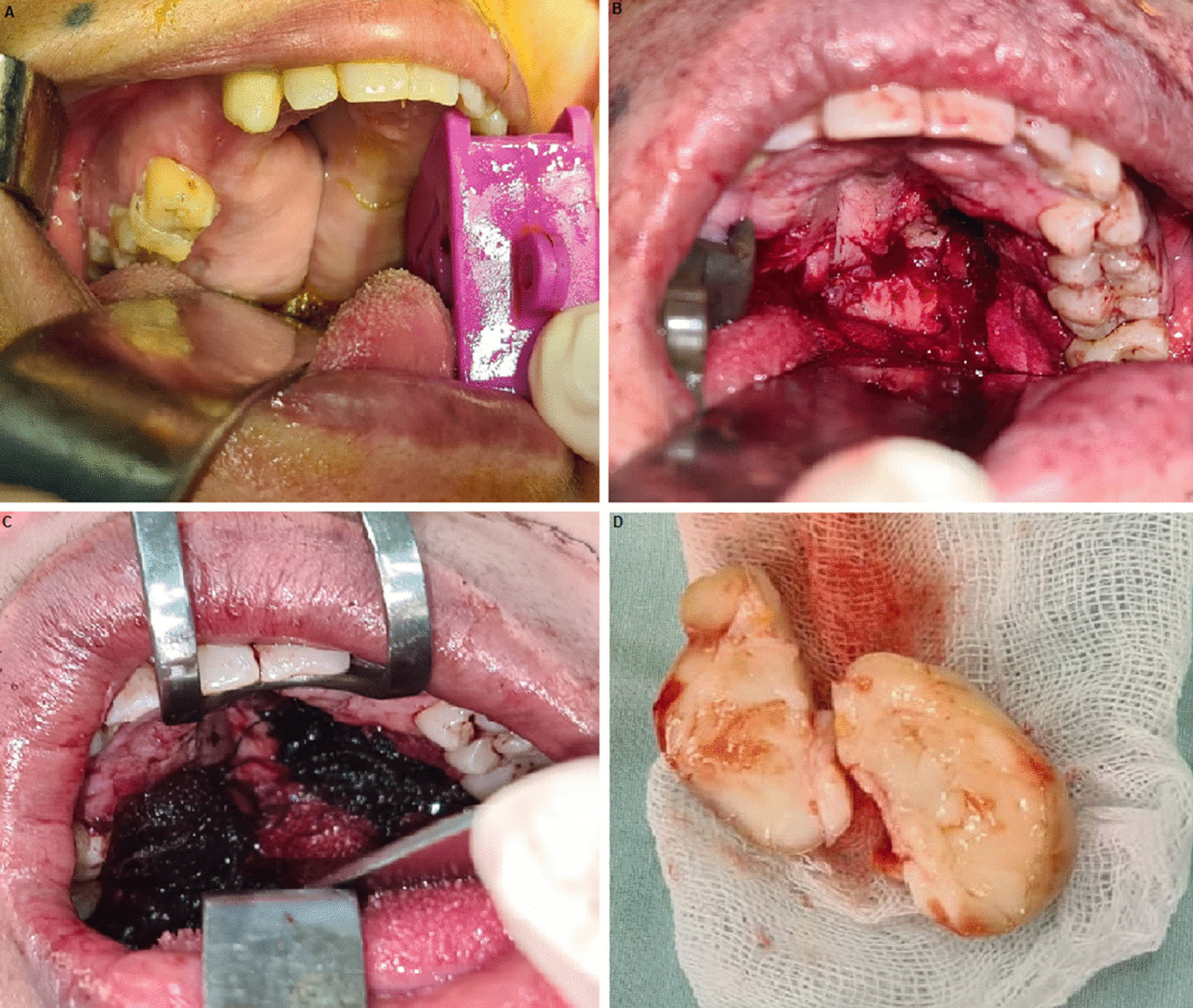
Surgical intraoperative pictures. **A** Preparation of the operation site. **B & C** Surgical site after excision. **D** Surgical specimen

Excisional biopsy was done under general anesthesia (Fig. 2A–D).

The two excised masses were fixed in 10% formalin solution and sent to the Oral Pathology Department in our faculty for histopathological examination.

On gross examination, the lesion was yellowish white and firm on cut surface. The specimens were embedded in paraffin blocks and stained using hematoxylin and eosin.

**Fig. 3:**
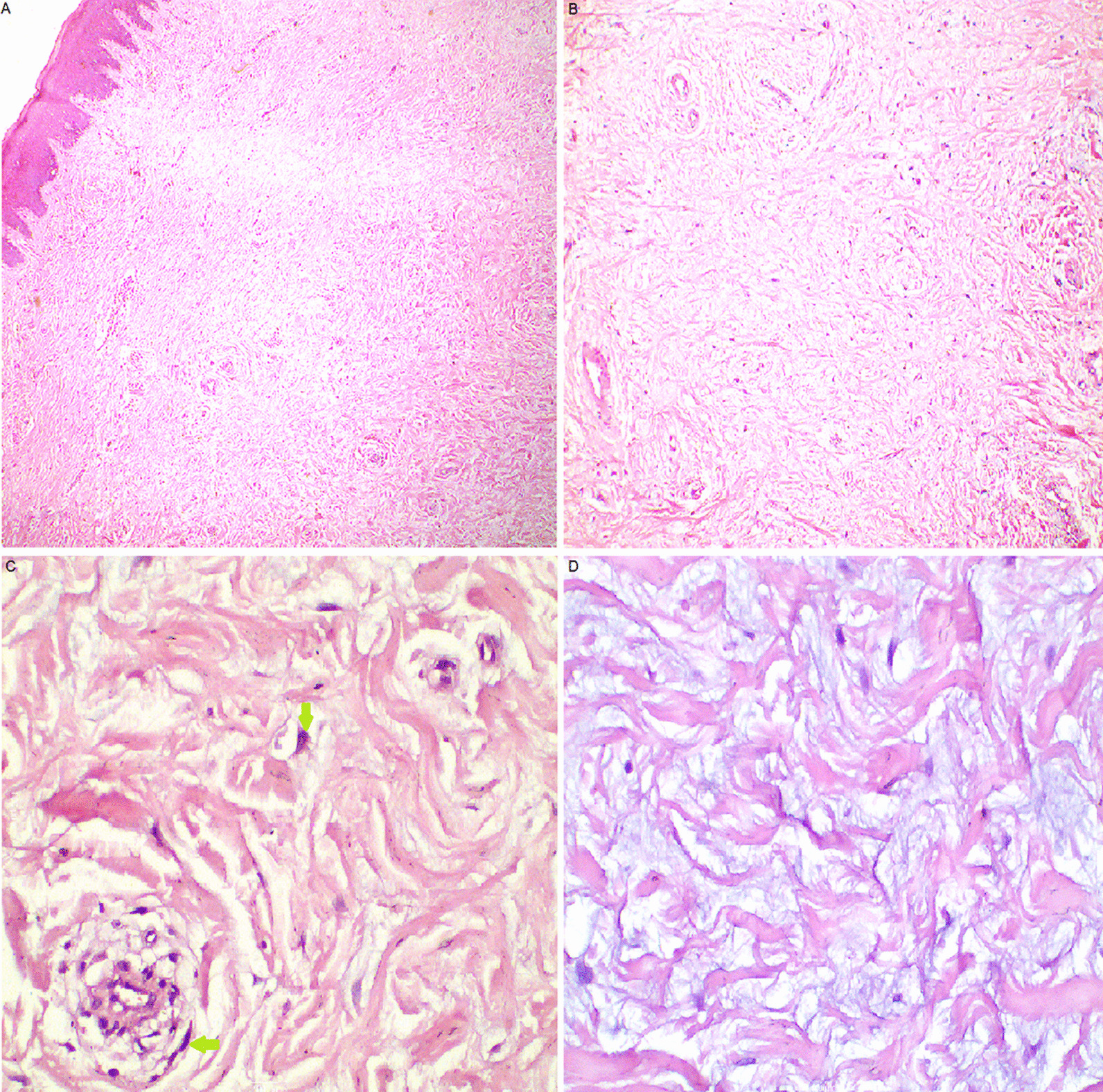
Histopathological features of the collagenous fibroma. **A** A connective tissue mass covered by keratinized stratified squamous epithelium (H&E* × 40). **B** Stellate and spindle- shaped cells in a heavily collagenized stroma with blood vessels (H&E × 100). **C** binucleated and trinucleated cells (green arrows) (H&E × 400). **D** Myxocollagenous stroma of the tumor (H&E × 400). *H&E* hematoxylin and eosin

Histopathological examination revealed a well-circumscribed connective tissue mass lined by keratinized stratified squamous epithelium (Fig. 3A). The connective tissue was heavily collagenized, formed of dense collagen bundles with sparsely distributed stellate and spindle-shaped cells (Fig. 3B). The nuclei were oval to elongated. Some cells were binucleated or trinucleated (Fig. 3C). No mitotic figures were present. Numerous blood vessels and few chronic inflammatory cells were noted. Myxomatous degeneration was detected in some areas of the stroma (Fig. 3D).

The histopathological picture suggested a benign mesenchymal tumor.

#### Final diagnosis

**Fig. 4:**
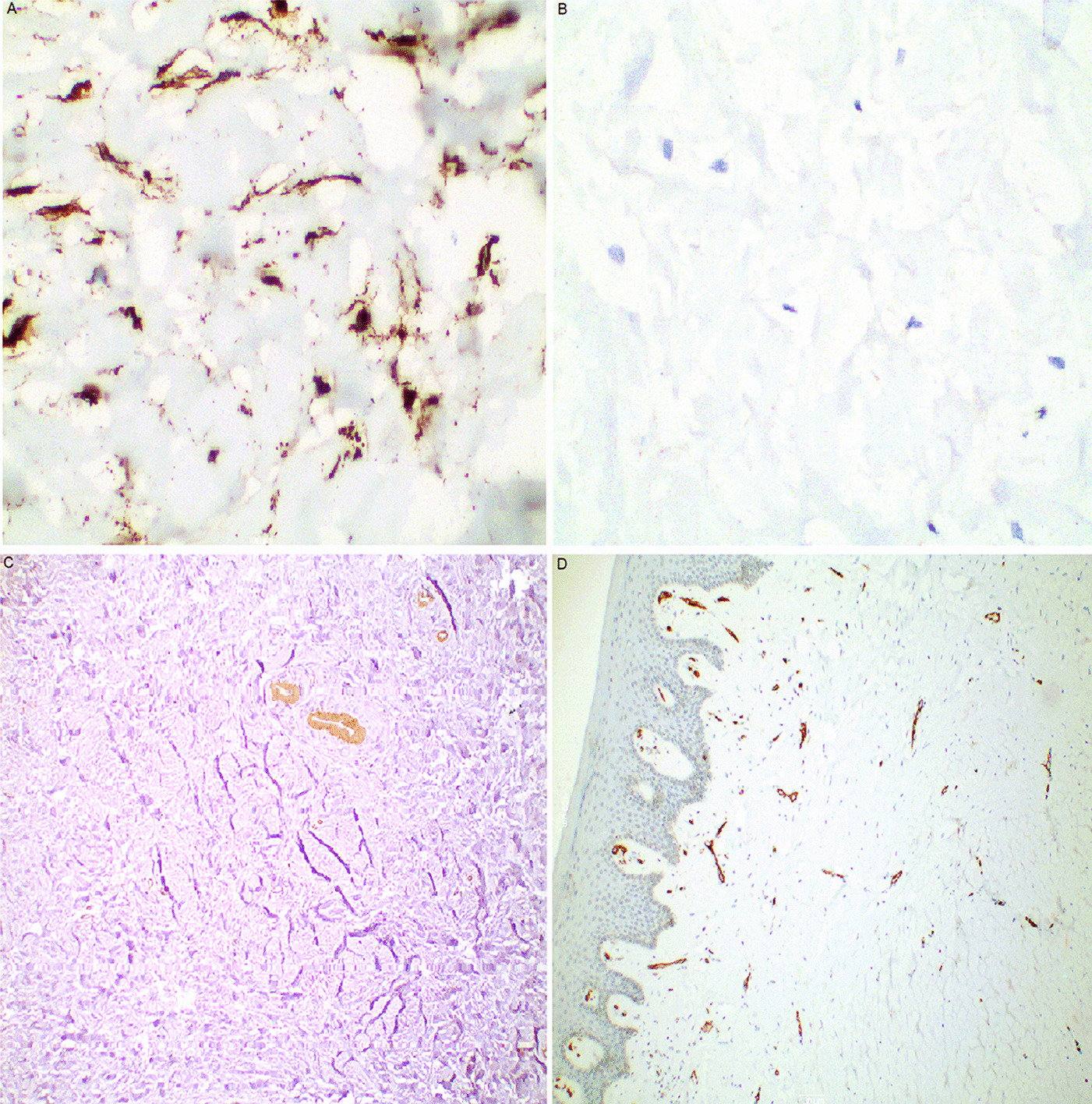
Immunohistochemical findings of the collagenous fibroma. **A** Tumor cells were strongly positive for vimentin (× 400). **B** Tumor cells were negative for beta-catenin (× 400). **C** Smooth muscle actin staining smooth muscle cells in blood vessels with no reaction in tumor cells (× 100). **D** CD34 staining endothelial cells of blood vessels with no reaction in tumor cells (× 100)

Immunohistochemical analysis revealed diffuse strong cytoplasmic staining of cells with vimentin (Fig. 4A). The cells were negative to pan cytokeratin, S-100, beta-catenin (Fig. 4B), smooth muscle actin (SMA), and CD34. SMA stained the smooth muscle cells in blood vessel lining (Fig. 4C) and CD34 showed positive staining of endothelial cells of the blood vessels in the tumor (Fig. 4D).

Diagnosis of bilateral collagenous fibroma was reached.

#### Follow-up

**Fig. 5:**
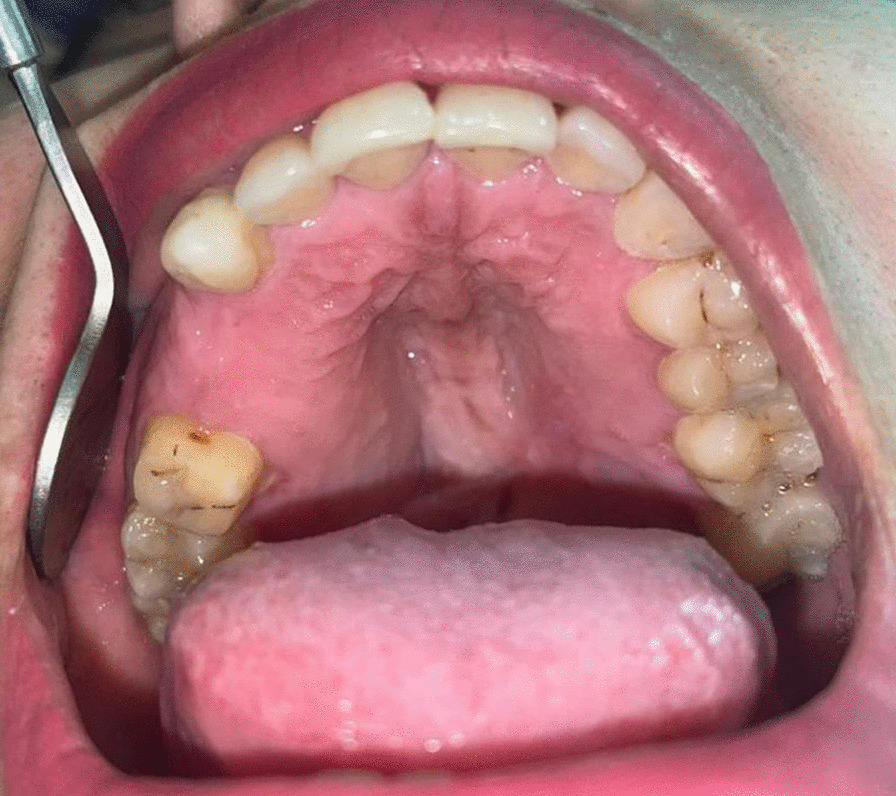
Six-month postoperative picture

After six months of surgical intervention, the patient came to the faculty hospital for follow-up. There was no sign of a developing lesion (Fig. 5). She was satisfied with the outcome of surgery and was feeling comfortable because of the removal of the huge mass that had been annoying her.

#### Review of literature

We reviewed the literature for collagenous fibroma. We searched for “collagenous fibroma in the oral cavity,” “desmoplastic fibroblastoma in the oral cavity,” and “bilateral collagenous fibroma” in PubMed and Web of Science databases. All English articles were selected. There were 15 articles and one of them was of a bilateral case.

Clinical data of the oral cavity cases are presented in Table 1.

**Table 1:** Clinical data of 15 cases of collagenous fibroma reported in the oral cavity

Author	Site	Size in cm	Age in years	Sex
Tokura *et al*., 2018 [3]	Tongue	1.3	66	F
Vasconcelos *et al*., 2018 [5]	Palate	4.5	48	F
Jaafari-Ashkavandi *et al*., 2018 [9]	Maxillary alveolar bone	2	58	F
de Sousa *et al*., 2011 [10]	Buccal mucosa	3.5	56	M
Garcia *et al*., 2018 [11]	Maxillary alveolar bone	2	43	F
Patil *et al*., 2015 [13]	Gingiva	2	45	F
Gonzales-Moles *et al*., 2004 [15]	Palate	2	55	F
Mesquita *et al*., 2001 [16]	Palate	5	37	F
Cazal *et al*., 2005 [17]	Maxillary alveolar ridge	1	42	F
Bhagalia *et al*., 2012 [18]	Gingiva	2	58	F
Pereira *et al*., 2016 [19]	Mandibular alveolar ridge	–	56	M
Shimoyama *et al*., 2005 [21]	Palate	6	49	F
Jham *et al*., 2013 [22]	Palate	3.5	56	F
Nonaka *et al*., 2010 [24]	Tongue	0.7	87	F
Varghese *et al*., 2014 [25]	Buccal mucosa	3	8	M

## Discussion

Collagenous fibroma was first identified and designated as desmoplastic fibroblastoma by Evans [6] in 1995, but the name collagenous fibroma was given by Nielsen *et al*. [7] due to its benign nature. It was suggested that it arises as a neoplasm rather than reactive proliferation because it is slowly growing and persistent with no preceding source of irritation [6–8]. In the head and neck region, the neck is the most affected site [8]. Few cases have been reported in the oral cavity [9]. The main patient complaint is discomfort when it reaches a large size, but the lesion is not painful [10]. Collagenous fibroma usually does not cause bony changes unless presented inside the bone, which is uncommon. Bony lesion presents as a well-defined radiolucency on radiographs [1, 9, 11].

Histopathologically, the tumor is hypocellular, formed of stellate and spindle-shaped fibroblasts and myofibroblasts embedded in a densely collagenized or myxocollagenized stroma [2]. This tumor shows some similarities with other lesions like desmoid tumor and focal fibrous hyperplasia. Yet, there are differences. Desmoid tumor is more cellular with high vascularity and infiltrative margins [3]. Focal fibrous hyperplasia shows inflammatory infiltrate and moderate cellularity [5].

Desmoid tumor or aggressive fibromatosis is an intermediate locally aggressive fibroblastic/myofibroblastic tumor. It has a high recurrence rate and causes destruction to the surrounding tissues. Treatment is by wide surgical excision [12]. In contrast to desmoid tumor, collagenous fibroma is a benign tumor that does not recur and is treated by conservative surgical excision with no need for grafts [1].

We present the second bilateral collagenous fibroma after the only reported case in English literature by Vasconcelos *et al*. [5].

Our case was a female patient having a mass in the hard palate. This is in accordance with most of the cases reported in the oral cavity, which were in female patients and were presented in the hard palate [5]. The ages of the cases reported in the oral cavity ranged from 8 to 87 years. Our patient’s age was within the same range: 37 years. The size of the current lesion was 5 cm in length. The reported cases had sizes ranging from 0.7 cm to 6 cm. Large lesions may cause difficulty in speech and mastication [10, 13]. Our case was complaining from the same problems. There was no history of trauma. This is in accordance with the results of many studies [4, 11, 13, 14], supporting the neoplastic nature of the lesion as suggested by Evans [6] and Nielsen *et al*. [7]. Miettinen and Fetsch [8] reported 2 cases with history of trauma out of 63 cases, making up the largest case series of collagenous fibroma.

The microscopic examination of the present case showed a well-circumscribed mass formed of stellate and spindle-shaped cells in a collagenous background. Almost all cases of collagenous fibroma have similar histologic findings [16–19]. However, nucleoli were not clearly seen as in the study of Gong *et al*. [1]. Blood vessels were quite numerous. This finding was opposite to the findings of other studies, where blood vessels were few [2–4, 14]. Despite the presence of binucleated and trinucleated cells, giant cell fibroma was not considered, since it usually measures less than 1 cm [20].

The histopathological findings were similar to focal fibrous hyperplasia; however, it was excluded due to large size of the lesion and absence of trauma. Focal fibrous hyperplasia usually does not exceed 1.5 cm [21]and is elicited by trauma [15].

Considering the bilateral nature of the present lesion, we considered desmoid tumor, which can be multiple in familial cases [12]. Yet, it was excluded due to the paucicellular nature of the current lesion, no interlacing of collagen bundles, and lack of infiltration at the margins.

In our case, there was infiltration of the surrounding fat tissue in accordance with the findings of Miettinen and Fetsch [8].

Immunohistochemistry is a very important diagnostic method that can help to reach a definitive diagnosis by excluding lesions, thereby guiding management. In the present case, the tumor cells were strongly positive to vimentin, supporting its fibroblastic origin. To differentiate this tumor from other tumors of different origins, we have done immunohistochemical staining by desmin, S-100 and CD34. There were negative reactions in cells, excluding muscle, nerve, and vascular tumors. Our results were similar to previous studies [5, 10, 14, 22]. In contrast to these studies, SMA staining was negative in our study. Some studies also showed negative SMA staining [9, 10, 23]. To rule out desmoid tumor, we did beta-catenin immunostaining [12]. Negative result favored the diagnosis of collagenous fibroma.

## Conclusions

Collagenous fibroma is a rare oral tumor that is to be considered in the differential diagnosis of fibrous proliferative lesions. Diagnosis can be reached by correlation between clinical picture, histopathological findings, and immunohistochemical results. Accurate diagnosis is necessary to avoid aggressive treatment.

## Declarations

### Availability of data and materials

Data sharing is not applicable to this article as no datasets were generated or analyzed during the current study.

## References

[1] Gong LH, Liu WF, Ding Y, Geng YH, Sun XQ, Huang XY (2018). Diagnosis and differential diagnosis of desmoplastic fibroblastoma by clinical, radiological and histopathological analysis. Chin Med J.

[2] Nakayama S, Nishio J, Aoki M, Nabeshima K, Yamamoto T (2021). An update on clinicopathological, imaging and genetic features of desmoplastic fibroblastoma (Collagenous Fibroma). In Vivo.

[3] Tokura T, Kobayashi J, Okamoto J, Miyazaki A (2018). Uncommon presentation of desmoplastic fibroblastoma on the tongue of a female patient. BMJ Case Rep.

[4] Nagaraja V, Coleman HG, Morgan GJ (2013). Desmoplastic fibroblastoma presenting as a parotid tumor: a case report and review of the literature. Head and Neck Pathol.

[5] Vasconcelos AC, Gomes AP, Tarquinio S, Abduch-Rodrigues E, Mesquita R, Silva K (2018). Oral bilateral collagenous fibroma: a previously unreported case and literature review. J Clin Exp Dent.

[6] Evans HL (1993). Desmoplastic fibroblastoma. A report of seven cases. Am J Surg Pathol..

[7] Nielsen GP, O’Connel JX, Dickersin GR, Rosenberg AE (1996). Collagenous fibroma (desmoplastic fibroblastoma): a report of seven cases. Modern Pathol.

[8] Miettinen M, Fetsch JF (1998). Collagenous fibroma (desmoplastic fibroblastoma): a clinicopathologic analysis of 63 cases of a distinctive soft tissue lesion with stellate-shaped fibroblasts. Human Pathol.

[9] Jaafari-Ashkavandi Z, Shirazi MY, Assar S (2018). Desmoplastic fibroblastoma in maxillary alveolar bone mimicking an odontogenic lesion: a novel case report with review of literature. Turk Patoloji Derg.

[10] de Sousa SF, Caldeira PC, Grossmann SD, de Aguiar MC, Mesquita RA (2011). Desmoplastic fibroblastoma (collagenous fibroma): a case identified in the buccal mucosa. Head Neck Pathol.

[11] Garcia NG, De Freitas Filho SA, Soares CT, Figueiredo CM, Oliveira DT. Intraosseous collagenous fibroma (Desmoplastic fibroblastoma) involving maxillary bone. J Clin Diagn Res. 2018;12.

[12] Goldblum JR, Folpe AL, Weiss SW. Enzinger and Weiss’s soft tissue tumors. Seventh Edition. Philadephia: Elsevier; 2020.9: borderline and malignant fibroblastic/myofibroblastic tumors. 306–374.

[13] Patil SR (2015). Intraoral desmoplastic fibroblastoma: a rare presentation. J Sci Soc.

[14] Ide F, Shimoyama T, Horie N, Tanaka H (1999). Collagenous fibroma (desmoplastic fibroblastoma) presenting as a parotid mass. J Oral Pathol Med.

[15] Gonzalez-Moles MA, Ruiz-Avila I, Gil-Montoya JA (2004). Collagenous fibroma (Desmoplastic fibroblastoma) of the palate associated with Marfan’s syndrome. Oral Oncol Extra.

[16] Mesquita RA, Okuda E, Jorge WA, de Araújo VC (2001). Collagenous fibroma (Desmoplastic fibroblastoma) of the palate: a case report. Oral Surg Oral Med Oral Pathol Oral Radio Endod.

[17] Cazal C, Etges A, de Almeida FCS, de Souza SCOM, Nunes FD, de Araújo VC (2005). Collagenous fibroma (desmoplastic fibroblastoma) of alveolar bone: a case report. J Bras Patol Med Lab.

[18] Bhagalia S, Jain M, Pardhe N, Sireesha SK (2012). Collagenous fibroma (desmoplastic fibroblastoma) of the oral cavity. J Oral Maxillofac Pathol.

[19] Pereira TSF, Lacerda JCT, Matias MDP, Jesus AO, Gomez RS, Mesquita RA (2016). Desmoplastic fibroblastoma (collagenous fibroma) of the oral cavity. J Clin Exp Dent.

[20] Neville BW, Damm DD, Allen CM, Chi AC (2016). Oral and maxillofacial pathology.

[21] Shimoyama T, Horie N, Ide F (2005). Collagenous fibroma (Desmoplastic fibroblastoma): a new case originating in the palate. Dentomaxillofac Radiol.

[22] Jham BC, Netto ACM, Resende RG, Ribeiro DC, Mesquita RA (2013). Pedunculated desmoplastic fibroblastoma (collagenous fibroma) of the oral cavity: a previously unreported clinical presentation. Int J Oral Maxillofac Pathol.

[23] Yang JH, Chae JB, Huh CH, Na JI, Park KC, Shin JW (2018). Desmoplastic fibroblastoma of the scalp accompanied by severe pain; unusual location and symptom. Ann Dermatol.

[24] Nonaka CFW, Carvalho MV, Moraes M, Medeiros AMC, Freitas RA (2010). Desmoplastic fibroblastoma (collagenous fibroma) of the tongue. J Cutan Pathol.

[25] Varghese T, Pillai KS, Sarojini SB, Khosla E (2014). Desmoplastic fibroblastoma (collagenous fibroma) in the oral cavity. J Indian Soc Pedod Prev Dent.

